# An early onset cone dystrophy due to *CEP290* mutation: a case report

**DOI:** 10.1007/s10633-023-09940-z

**Published:** 2023-08-29

**Authors:** Anastasia Binder, Susanne Kohl, Ute Grasshoff, Karin Schäferhoff, Katarina Stingl

**Affiliations:** 1 Augencentrum Südwest, Seestrasse 59B, 70174 Stuttgart, Germany; 2 Institute for Ophthalmic Research, Centre for Ophthalmology, University of Tübingen, Tübingen, Germany; 3 Institute of Medical Genetics and Applied Genomics, University of Tübingen, Tübingen, Germany; 4 University Eye Hospital Tübingen, Centre for Ophthalmology, University of Tübingen, Tübingen, Germany; 5 Center for Rare Eye Diseases, University of Tübingen, Tübingen, Germany

**Keywords:** *CEP290*, Cone dystrophy, Achromatopsia, Ciliopathies

## Abstract

**Purpose:**

Biallelic mutations in the *CEP290* gene cause early onset retinal dystrophy or syndromic disease such as Senior-Loken or Joubert syndrome. Here, we present an unusual non-syndromic case of a juvenile retinal dystrophy caused by biallelic *CEP290* mutations imitating initially the phenotype of achromatopsia or slowly progressing cone dystrophy.

**Methods:**

We present 13 years of follow-up of a female patient who presented first with symptoms and findings typical for achromatopsia. The patient underwent functional and morphologic examinations, including fundus autofluorescence imaging, spectral-domain optical coherence tomography, electroretinography, color vision and visual field testing.

**Results:**

Diagnostic genetic testing via whole genome sequencing and virtual inherited retinal disease gene panel evaluation finally identified two compound heterozygous variants c.4452_4455del;p.(Lys1484Asnfs*4) and c.2414T > C;p.(Leu805Pro) in the *CEP290* gene.

**Conclusions:**

*CEP290* mutation causes a wide variety of clinical phenotypes. The presented case shows a phenotype resembling achromatopsia or early onset slowly progressing cone dystrophy.

## Introduction

Inherited retinal diseases (IRD) are a clinically and genetically heterogenous group of conditions with dysfunction and/or degeneration of cells of the retina. Since cilia are essential for many cell types including photoreceptors, there is a wide range of retinal phenotypes associated with mutations in genes affecting the photoreceptor connecting cilium. Resulting diseases are called ciliopathies [1]. One of the most intriguing ciliopathy-associated disease genes is the gene for Centrosomal Protein 290 (*CEP290*), in which mutations cause various distinct retinal phenotypes, ranging from non-syndromic Leber congenital amaurosis, early onset IRD or retinitis pigmentosa over Senior-Loken, Joubert, and Bardet-Biedl, to lethal Meckel-Grüber syndrome [1, 2]. The *CEP290* gene encodes a protein involved in ciliary assembly and trafficking. It is expressed in photoreceptors, but also in multiple other tissues. Today, more than 100 *CEP290* mutations have been described, but the phenotype–genotype correlation is not fully understood [1]. In photoreceptors, *CEP290* localizes to the connecting cilium, the transitional zone linking the inner and outer segments of rods and cones [3]. The most typical presentation of *CEP290* intraretinal disease seems to be Leber congenital amaurosis (LCA), followed by retinitis pigmentosa and cone–rod dystrophy [4]. The LCA is the earliest and most severe form of all inherited retinal dystrophies, causing profound visual deficiency, nystagmus, and an undetectable or severely reduced ERG in the first year of life. [1] Retinitis pigmentosa or severe cone–rod-type retinal dystrophy phenotypes caused by *CEP290* lead usually to severe visual impairment. Visual field deteriorate relatively fast and electroretinograms are undetectable in the majority of the patients [4]. Early fundus changes include white dots or a marbleized or salt and pepper aspect and progress to midperipheral nummular or spicule pigmentation [5]. However, milder cases with almost normal visual acuity in adult age are known, too [4, 5],

A detailed study of the retinal architecture of human *CEP290*-mutant retinas identified profound retinal remodeling in the peripheral rod-rich regions and no clear alterations in the cone-rich foveal region. This difference in rod and cone degeneration may point to a distinct function of *CEP290* in both cell types [6].

In this report, we present a patient with cone-dominated IRD carrying biallelic heterozygous variants in *CEP290*, showing characteristics of achromatopsia, thereby broadening the spectrum of *CEP290*-associated disease.

## Case presentation

A female patient of Caucasian origin presented at the University Eye Hospital Tübingen, Germany at 6 months of age with nystagmus which persisted since birth. No other developmental abnormalities were observed.

Ophthalmologic examination showed pendulum-shaped, rotational and also dissociated nystagmus beating in all directions. Additionally, the patient showed frequent rubbing of the eyes, especially when tired. At the age of 2 years, she also developed increased glare sensitivity and slight anisocoria. Visual acuity was always poor, and retinoscopy showed bilateral age-related hyperopia of ~ 3 diopters at the age of 2 years. Later, the patient developed divergent strabism with left eye fixation. Funduscopy showed normal retina, the remaining morphology of the anterior eye segment did not show any pathologies.

**Fig. 1:**
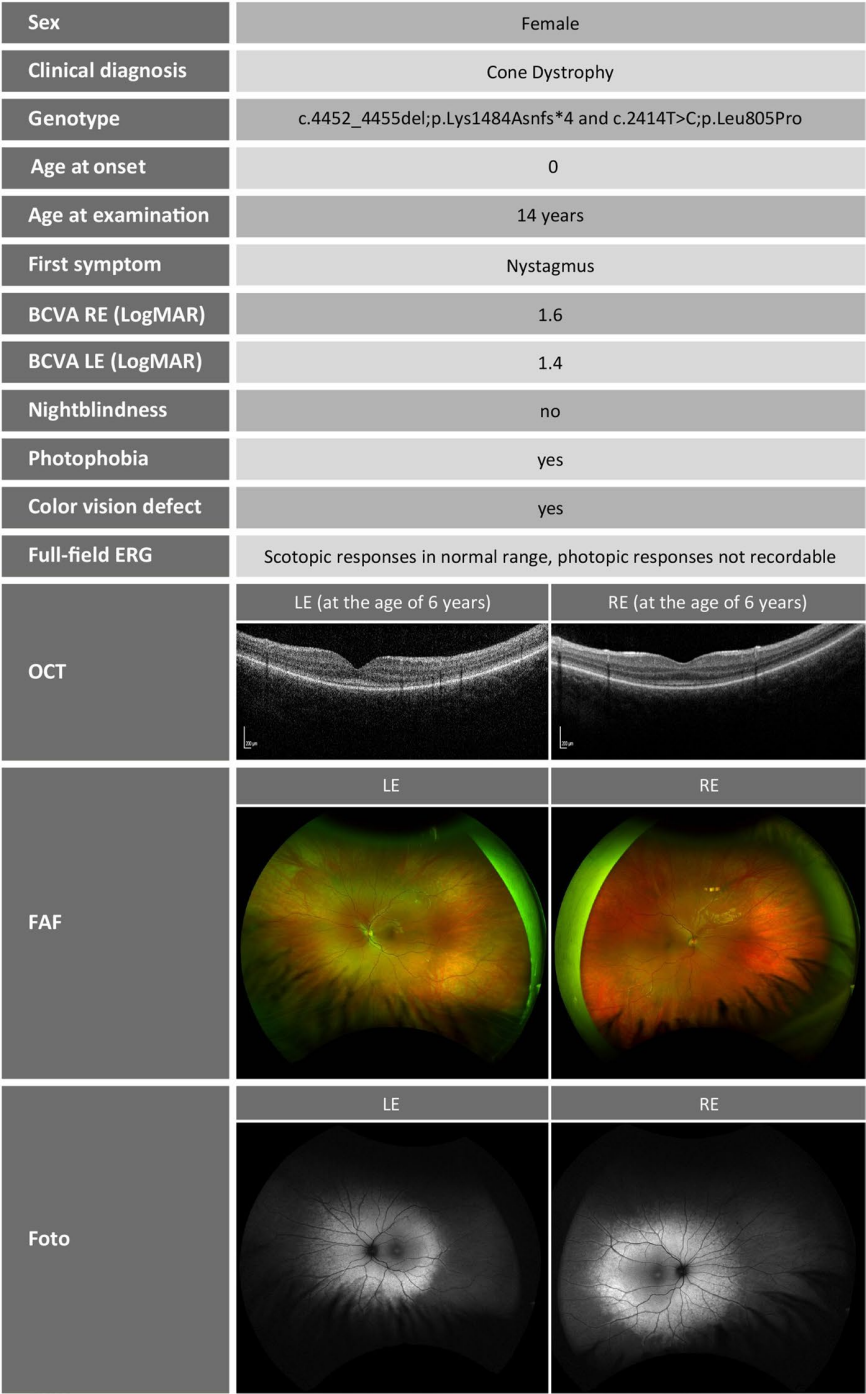
Overview of the clinical findings of the patients. BCVA, best corrected visual acuity; RE, right eye; LE, left eye; ERG, electroretinogram; OCT, optical coherence tomography; FAF, fundus autofluorescence

**Fig. 2:**
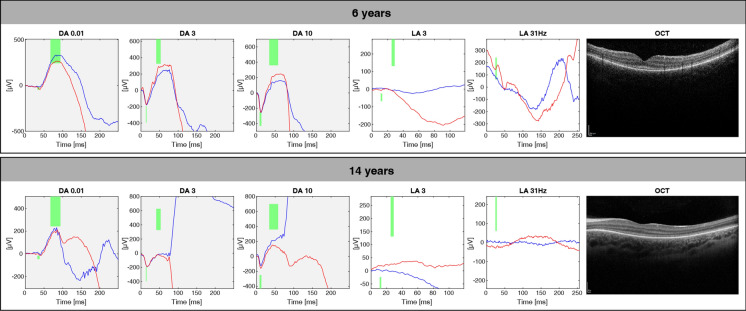
Full-field electroretinography was performed at the age of 6 years and 14 years according to the ISCEV (international society for electrophysiology of vision) standards. Due to strong photophobia, some of the recordings are biased by lid and movement artifacts, but demonstrate a non-recordable light-adapted response at both visits and close-to-normal dark-adapted responses. The corresponding OCT images on the right show macular layering with a diffuse atrophy of the outer segments. The Fundus photographs and Autofluorescence of both eyes show central thinning of the retina which presents as a lighter appearance of the central part of the retina. DA,  dark-adapted; LA, light-adapted; traces in red, right eye; traces in blue, left eye; green areas, age-adapted norm values

Multimodal diagnostics of the retina was performed at the age of 6 years. The best corrected visual acuity (BCVA) was logMAR 1.6 on the right and 1.4 on the left eye. In the visual field (semiautomated kinetic perimetry), a slight concentric constriction and a central scotoma were seen; however, due to the young age, this could have been caused by reduced cooperation during perimetry, or by nystagmus. Photophobia persisted. Optical coherence tomography (OCT) showed macular layering with a slight diffuse atrophy of the outer segments (Fig. 1). Full-field electroretinography (ERG) at the age of 6 years showed normal scotopic responses, but abnormal shape of the oscillatory potentials and no reproducible photopic responses (Fig. 2). The Farnsworth test performed binocularly revealed color perception disorder along all three axes and anomaloscope examination suggested findings consistent with achromatopsia.

Despite repeated and extensive genetic analysis for mutations in upcoming known achromatopsia genes, no causative mutations were detected.

At the age of 14 years, the clinical findings did not reveal much progression (Fig. 1). Wide-field fundus autofluorescence performed at the age of 14 years showed a hypo- and hyperautofluorescent pattern (Fig. 1) not typical for achromatopsia.

Finally, IRD gene analysis out of whole genome sequencing data within a diagnostic genetic testing setup at the age of 14 years identified two heterozygous variants: c.4452_4455del;p.(Lys1484Asnfs*4) and c.2414T > C;p.(Leu805Pro) in *CEP290* (ENST00000552810.1). Segregation analysis in both parents confirmed biallelic occurrence (Fig. 2).

The variant c.4452_4455del;p.(Lys1484Asnfs*4) has already been associated with *CEP290*-associated phenotypes [2, 7, 8]. It results in frame shift and a truncated protein; the transcript is predicted to undergo nonsense mediated decay. It is classified as pathogenic. The variant c.2414T > C;p.(Leu805Pro) has also been reported in the literature and ClinVar (https://www.ncbi.nlm.nih.gov/clinvar/variation/866922/) is also associated with *CEP290*-related diseases [2]. This missense variant affects an evolutionary highly conserved amino acid residue in the coiled-coil domain CC IV of the SMC (structural maintenance of chromosome) homology domain of *CEP290* [9]. Yet, prediction software ratings are inconsistent and classify this variant as variant of uncertain significance.

In the pedigree, no further family members were affected with a *CEP290* phenotype or other type of inherited retinal degeneration.

## Discussion

*CEP290* mutations are associated with autosomal recessively inherited early childhood IRDs. Typically, the *CEP290*-related phenotype presents an early onset retinal dystrophy or LCA, or a severe cone–rod-type retinal dystrophy [4]. The *CEP290* phenotype covers approximately 30% of patients with the typical LCA, whereas the most common variant is the deep intronic mutation c.2991 + 1665 [4]. In most of the cases, the *CEP290* phenotype is characterized by an early onset of problems associated with rod and cone dysfunction, such as nystagmus, night blindness, visual field defect and reduced vision with a relatively rapid progression [4]. Still, cases of a typical retinitis pigmentosa with later onset and slower progression have been reported, too [4, 5]. The phenotype spectrum of *CEP290* related retinal dystrophies is rather broad and includes also syndromic diseases such as Senior-Loken syndrome, Joubert syndrome, Bardet-Biedl syndrome or Meckel-Grüber syndrome [1, 2]. De Baere et al. described over 100 different mutations in *CEP290* [1]. Resulting phenotypes are variable and can involve single or multiple organs. For the majority of mutations, no clear-cut genotype–phenotype correlation could be established.

Here, we describe an atypical case of a slowly progressing cone dystrophy caused by biallelic *CEP290* variants, initially diagnosed as achromatopsia. Such a phenotype with early onset of visual impairment with nystagmus and photophobia demonstrating cone dysfunction from early age on; however, very slow progression up to the age of 14 years has not been published in the spectrum of *CEP290* phenotypes to our knowledge yet. Due to this clinical presentation of barely progression cone dysfunction, the case was initially held for achromatopsia.

Achromatopsia is an inherited retinal disease with absent cone function resulting in congenital nystagmus, low vision, photophobia and inability to distinguish colors [10–12]. Patients with incomplete achromatopsia have a remaining cone function and present usually with milder symptoms. In its clinical presentation, it is mostly a stationary disease, but due to modern high-resolution imaging such as OCT, we know that there are progressive degenerative changes of cones in achromatopsia [13–15]. The initial presentation of achromatopsia is usually in the first month after birth with a life-long reduced visual acuity around 20/200 in the case of complete achromatopsia. In electroretinography, the cone signals are undetectable, or reduced in some cases of incomplete achromatopsia [13]. Based on clinical presentation, also other very slowly progressing cone dystrophies can be held for achromatopsia [16].

In our patient, the *CEP290* variants lead to an early onset nystagmus, photophobia and low vision, along with unremarkable funduscopy, which clinically at first seemed consistent with achromatopsia. Additionally, during the first years, no clear progression could be documented. Moreover, the full-field electroretinography at the age of 6 showed undetectable cone responses with normal rod responses, which additionally supported the diagnosis of achromatopsia. Later, the diagnosis was corrected to cone dystrophy, especially based on the atypical fundus autofluorescence pattern that did not match to retinal imaging known for achromatopsia. Usually, patients with achromatopsia present with a normal or almost normal fundus autofluorescence with minimal foveal and perifoveal changes, thus such an autofluorescence pattern as in this case would be atypical for achromats [13].

Finally, by means of a genome sequencing, it could be shown that the case is a *CEP290* phenotype, probably a cone dystrophy with a very slow progression.

Complete loss of function of both *CEP290* alleles typically leads to Joubert syndrome, whereas patients with Leber congenital amaurosis and early onset IRD are expected to have a small amount of residual *CEP290* activity [9].

*CEP290*-related IRD typically presents with congenital nystagmus or roving eye movements and hyperopia [6]. Both were also observed in our patient. Also, the severely reduced but not progressing loss of BCVA was reported earlier [17].

Swaroop et al. also described retinas of blind *CEP290*-related IRD patients did not show central degeneration and thinning [6]. This resembles our patients’ findings with only slight changes in the outer retinal layers and severe BCVA impairment despite normal central retinal structure. The central retina of the *CEP290* phenotypes shows slowly progressive degeneration [6, 17]. In particular, the ellipsoid zone band width declined at a mean rate of about  −2%/year [17]. However, these progression signs were not present in the first 14 years of our patient.

Also, Michaelides et al. previously described younger subjects are more likely to have a normal fundus, while older patients commonly showed evidence of peripheral retinal pigment migration [17, 18]. In addition, in our case, there is a discrepancy of well recordable scotopic ERG and a non-recordable photopic ERG in combination with a non-progressive IRD misleading the clinical diagnosis initially to achromatopsia, while typically *CEP290*-related IRD is associate with impaired or undetectable rod and cone responses [17].

The presented case expands the known spectrum of CPE290 related retinal disease. Long-term observation of this young patient is necessary to understand unusual genotype–phenotype correlations.

## Conclusions

We present a patient with cone-dominated IRD, resembling initially achromatopsia and carrying biallelic compound heterozygous variants in *CEP290*, broadening the spectrum of *CEP290*-associated disease.
